# Effect of the Combination of Restorative Material and the Etching Protocol in Enamel Microleakage in Class II Cavities after Thermocycling

**DOI:** 10.1155/2023/1354738

**Published:** 2023-02-11

**Authors:** Natalia Menezes Santana Dutra, Kusai Baroudi, Aline Spagnol Fedoce Silva, Laísa Araujo Cortines Laxe, Laís Regiane da Silva Concilio, João Paulo Mendes Tribst, Luciana Andrea Salvio

**Affiliations:** ^1^Department of Restorative Dentistry, School of Dentistry, Federal University of Juiz de Fora, Juiz de Fora, Brazil; ^2^RAK College of Dental Sciences, RAK Medical & Health Sciences University, Ras Al Khaimah, UAE; ^3^Postgraduate Program, School of Dentistry, University of Taubate, Taubate, Brazil; ^4^Department of Oral Regenerative Medicine, Academic Centre for Dentistry Amsterdam (ACTA), Universiteit van Amsterdam and Vrije Universiteit, 1081 LA Amsterdam, Netherlands

## Abstract

This study is aimed at evaluating the marginal microleakage of bulk-fill class II restorations after thermocycling. Thirty-two human third molars received class II cavities prepared on mesial and distal faces. The cavities were bonded according to the adhesive protocol (total or self-etching). The cavities were then restored with composite and divided into 8 groups based on the composite combination: layering technique with Filtek Z350XT (G1 and G5), first layer with Filtek Z350 XT Flowable Restorative and then Filtek Z350XT (G2 and G6), bulk-fill technique with Filtek One Bulk Fill (G3 and G7), and first layer with Filtek One Bulk Fill Flow and Filtek Z350 XT (G4 and G8). The restorations were then subjected to thermocycling (2000 cycles, 5°C and 55°C, dwell time of 30 s). After aging, the restored teeth were immersed in methylene blue for 48 hours. The restorations (*n* = 32) were sectioned in the middle (two sections). The microleakage results were analyzed by two-way ANOVA followed by Tukey's post hoc test (*α* = 0.05). The groups did not differ statistically from each other in terms of marginal microleakage (*p* = 0.295). It can be concluded that there is no difference in the percentage of microleakage between conventional and bulk-fill resins on both consistencies, as well as there is also no statistically significant difference when the adhesive system is used in a conventional or self-etching mode.

## 1. Introduction

Light-cured composite resins, also called dental composites, have been in constant development [1]. Advances in chemical composition, stronger inorganic particles, and adhesive techniques resulted in modified and improved dental materials [2]. It is known that the success of composite resin restoration depends on adequate polymerization of the organic matrix and the conversion of monomers to polymers [3]. A process that generates polymerization shrinkage stresses can result in marginal gap, enamel fractures, and cusp deflections with consequent microleakage, postoperative sensitivity, and secondary caries [3, 4]. Therefore, different restorative techniques and types of composite resins are used to minimize these problems.

To reduce the stresses caused by polymerization shrinkage during composite placement, a technique known as the “incremental technique” is used [5, 6]. This is a very sensitive technique and requires considerable clinical time from the operator [7]. The bulk-fill composite resins were developed to minimize chair time and technique sensitivity. They can be used in whole increments from 4 to 6 mm [8, 9] and clinical time savings [10]. Currently, bulk-fill resins are available in flowable and regular viscosity [3, 11]. Both can be polymerized in increments of 4 mm or more depending on the trademark. However, the flowable version may require a final resin layer of conventional viscosity to create anatomical features [3]. In addition, bulk-fill flowable composite resin has a low modulus of elasticity and lower hardness. Therefore, the wear rate of flowable composite is higher compared to conventional composite and requires coverage [12]. On the other hand, low viscosity bulk-fill resins do not require another material combination once they can completely fill the cavity [13].

The main advantage of bulk fill resins is the reduction of work time, especially for large cavities [13]. Manufacturers claim that bulk-fill composites have a greater depth of cure [14]. This may be due in part to the presence of a certain type of photoinitiator and greater translucency compared to conventional composites [6, 11]. This was only possible with new photoinitiators and increased translucency [15, 16].

For certain types of bulk-fill resins, there was a decrease in the amount of inorganic particles to achieve high translucency, which justified the reduced mechanical properties compared to conventional composite resins [11, 17]. According to the literature [3], a lack of consensus is still present in relation to these materials, which discourages their use in deep cavities. Additionally, there are reports that bulk-fill technique can still cause unpolymerized particles and free monomers on the restoration causing toxicity in the adjacent tissue [15].

The aim of the present study was to evaluate the marginal microleakage of bulk-fill and conventional class II restorations after thermocycling. The null hypothesis was that there is no difference in microleakage between bulk-fill and conventional restorations in both viscosities.

## 2. Materials and Methods

This in vitro study was developed at the Integrated Dental Research Laboratory of Faculty of Dentistry of Federal University of Juiz de Fora after approval under number 2.692.137 by the Human Research Ethics Committee of same institution. Thirty-two healthy human teeth belonging to the third molar group were donated by the UFJF Human Teeth Bank.

### 2.1. Teeth Preparation

The occlusal surface of each tooth was flattened using sandpaper and water-cooling. The teeth were embedded in chemically activated acrylic resin using a metallic matrix to standardize the occlusal surface positioning. Removal of the occlusal enamel was necessary to provide a flat surface to fix the light-curing unit device (Bluephase, Ivoclar-Vivadent) repeatedly at the same distance (2 mm) from the occlusal margin.

**Figure 1:**
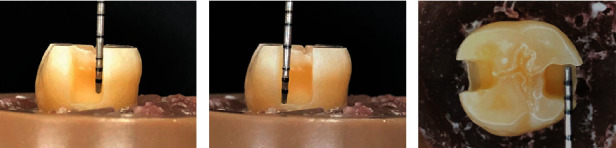
Proximal view of the cavity preparation: (a) millimeter probe indicating the depth of the pulp wall (4 mm), (b) millimeter probe indicating the depth of the gingival wall (5 mm), and (c) occlusal view of the tooth and the mesial and distal cavities.

**Figure 2:**
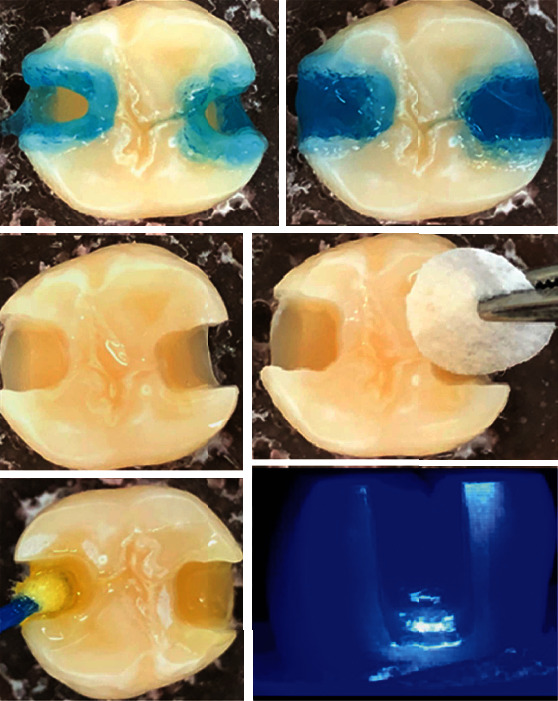
Total etching protocol performed in groups 1-4.

Two class II proximal boxes with 4 mm (±0.2 mm) deep on the pulp wall, 5 mm (±0.2 mm) on the gingival wall, and 2 mm (±0.2 mm) width were prepared using a cylindrical diamond drill (number 1092, KaVo Brazil, Joenville, SC) at high speed and under refrigeration. The dimensions of each cavity were measured with a dental probe (Quinelato, Schobell Industrial Ltda, Rio Claro, SP, Brazil). The coronary and gingival margins were necessarily located in enamel (Figures 1 and 2).

### 2.2. Restorative Procedures

The prepared teeth were randomly distributed in eight groups (*N* = 8). The cavities were restored with composite Filtek One Bulk Fill (FBP), Filtek Bulk Fill Flow (FBF), Filtek Z350XT (Z350), and Filtek Z350XT Flowable (ZF) composites (A2, 3 M ESPE St. Paul MN, USA all composites) along with Single Bond Universal Adhesive (3 M ESPE St. Paul MN, USA) in full etching or self-etching modes by a single operator. The composite materials are summarized in Table 1. All restorations were light-cured as recommended with the aid of LED device (Bluephase, Ivoclar-Vivadent) with a power of 800 mW/cm^2^.

### 2.3. Etching Protocol

Half of the specimens (groups 1-4) received the adhesive system with total acid etching: 37% phosphoric acid for 30 s in enamel and 15 s in dentin and subsequent removal with water jet for 30 s, drying with absorbent paper, keeping the dentin moist and the enamel dry, and applying a single layer of SBU adhesive (1 drop) actively throughout the cavity using a microbrush (Figure 2).

**Figure 3:**
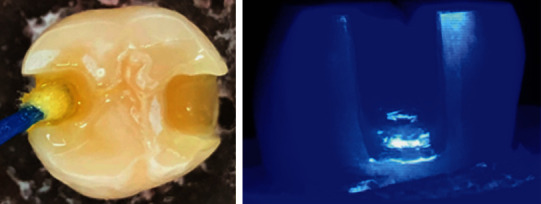
Self-etching protocol performed in groups 5-8.

The other half of the specimens, (groups 5-8), received a self-etching adhesive, applying a single layer of SBU adhesive (1 drop) actively throughout the cavity using a disposable brush (Figure 3).

**Figure 4:**
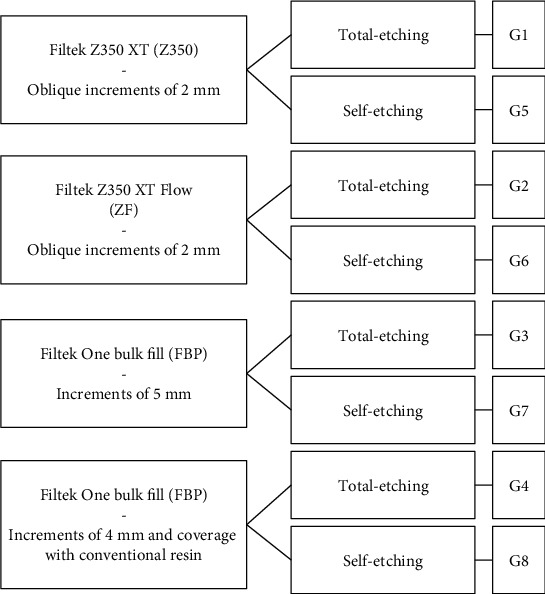
Flowchart with the study design according to different restorative procedures and etching mode.

For both protocols, the solvent was volatilized for 5 s, and the light curing was carried out for 10 s keeping the tip of light curing-unit at 2 mm from the cavity with standardized distance.  Figure 4 summarizes the study design.

After the restorative procedure, the occlusal and gingival margins of the restorations were finished with flexible polishing disks of decreasing granulation (Soflex Pop-On, 3 M ESPE, St. Paul, USA). Then, the samples were stored in deionized water at 37°C for 24 hours. At the end of this period, the samples were subjected to thermal aging with 2000 cycles (5°C and 55°C—dwell time of 30 s).

### 2.4. Microleakage Evaluation

The specimen root was sealed with chemically activated acrylic resin and covered with two layers of cosmetic varnish (Revlon enamel, red color, Rio de Janeiro-RJ, Brazil) on all dental surfaces, except for 1 mm below restoration gingival margins. They were stored in a 2% methylene blue solution (Santos Homeopathy, Juiz de Fora-MG, Brazil) for 48 hours at 37°C). After 48 hours, the teeth were washed to remove excess dye. The teeth were then sectioned with a diamond disk in a precision cutting machine (Isomet, Buehler Ltda, Lake Bluff, IL). The analysis of penetration of the dye into the dental substrate was performed using ImageJ software, where the values of total length of the bonding interface and extent infiltrated by the dye were obtained.

### 2.5. Statistical Analysis

The data were measured by the same examiner at two different times (one-week interval) to check the intraexaminer reliability index, using the intraclass correlation coefficient test. Data were analyzed for normality using the Kolmogorov-Smirnov test and for homoscedasticity using the Levene's test. Two-way analysis of variance was used to assess the effects and interaction of the factors “composite resins” and “type of hybridization” in microleakage. Data were analyzed using the SPSS 22 software, and the level of significance adopted was 5%.

## 3. Results

The intraclass correlation coefficient test showed an excellent intraexaminer correlation (0.99). The data from the first analysis was used to perform the other statistical tests. The data showed a homogeneous distribution (*p* = 0.05), and groups 2 (*p* = 0.20) and 3 (*p* = 0.20) showed a normal distribution.

The average values and the standard deviation of the percentage of microleakage observed in the tooth-composite resin interface used are described in Table 1. The factors “composite resins” (*p* = 0.051) and “etching mode” (*p* = 0.172) did not differ statistically, as well as the interaction between these factors (*p* = 0.295). The descriptive statistics are summarized in Table 2.

There was no statistical difference between the groups, and the etching mode did not interfere with the microleakage of the composite resins (Table 3).

## 4. Discussion

The present in vitro study evaluated the microleakage of class II restorations with different composites after thermos cycling. The results showed that there is no statistically significant difference between the groups evaluated; therefore, the null hypothesis was accepted.

Marginal microleakage is still a major drawback of dental composite restorations, which is mainly caused by the polymerization shrinkage and increased by thermal fatigue in the oral environment [18]. Marginal gap contributes to microleakage, allowing the passage of fluids and bacteria from the oral cavity as a source of postoperative sensitivity, pulp inflammation, and recurrent caries [19]. Dental restorations are subject to constant aging in the oral environment, such as changes in temperature and pH. Artificial aging, through thermocycling, favors the marginal microleakage of resin restorations as a reproducible method to test the sealing ability of the restorative material at artificial aging [18].

The temperature variation induces stresses that can lead to the formation of gaps and microleakage at the adhesive interface, resulting from the differences between the thermal expansion coefficients of the restorative material and the natural tooth. Previous studies have shown that thermocycling is the most effective method to simulate the microleakage process [20]. Based on that, the present investigation performed 2000 thermal cycles between 5 and 55°C.

The link between the tooth tissue and the restorative material is the critical region of the adhesive interface; and failures in this interface can lead to microleakage. Therefore, through acid etching, collagen fibrils can be exposed and infiltrated with hydrophilic and hydrophobic monomers creating a hybrid layer with micromechanical union. In self-etching adhesive systems, when used in dentin, some amounts of hydroxyapatite remain around collagen fibrils due to the low acidity of the acid monomer, favoring chemical union with the functional monomer and improving bond strength [18]. The self-etching in the enamel tissue causes mild demineralization with the formation of calcium salts, resulting in a weak chemical bond with hydroxyapatite [21]. Therefore, selective enamel etching on the surface of the cavity would provide a better marginal seal [20]. In the present study, the universal adhesive system was used in a conventional manner, etching enamel, and dentin with 37% phosphoric acid.

Dental composite resins generally have two distinct phases: an organic matrix composed mainly of resinous monomers such as bisphenol-A glycidyl methacrylate (BisGMA) and an inorganic phase composed of quartz or aluminosilicate particles [22]. These phases are chemically joined by bonding agents (silane), which allow the transfer of forces from the organic matrix to the inorganic particles, which then favors the mechanical properties and chemical stability of the material. However, even with the numerous improvements in the restorative material properties, some problems still remain such as the polymerization shrinkage stress [23]. This residual stress is inherent to the material and occurs due to the contraction of monomers during polymerization [24]. The magnitude of the stress is highly dependent on the properties of material [25]; however, if the stress transmitted to the tooth-restoration interface exceeds the bond strength, joint failures are formed [26].

Techniques to minimize the stresses created by polymerization shrinkage have been developed [25, 27], and, in that sense, the incremental technique has become widely popular [25]. In the other hand, some bulk-fill resins present a significant reduction in the shrinkage stress while maintaining adequate polymerization at a thickness of 4 mm. This may justify the use of these materials in cavities with high C-factor and deep cavities [28]. The appropriate degree of conversion is clinically significant because it affects the RC hardness, modulus of elasticity, dimensional stability, solubility, water absorption, color stability, and biocompatibility [29]. In the present study, the regular bulk fill resin proved to be inferior to conventional resin with regard to microleakage (Tables 2 and 3), suggesting deficiency in polymerization throughout the length of cavity and polymerization shrinkage of the material. Insufficient polymerization can lead to a decrease in the physical, mechanical, and biological properties of composite resins [30].

Two different viscosities of bulk-fill resins are available, allowing to classify them between regular and flowable. Both have different mechanical properties and can share or not the same clinical indication [31]. In this way, bulk-fill resins with flowable consistency can be used as a base to better adapt to the rounded angles of the cavity. High-viscosity resin composites can be used to restore the missing tissue [32], but they have a difficult adaptation in the cavity, which compromises the adequate marginal seal in comparison with low-viscosity materials [20]. According to the literature, flowable RC has a coefficient of expansion close to that of the tooth and a reduced coefficient of elasticity, which decreases their polymerization shrinkage and thereby decreases microleakage [33]. However, according to the present study, there was no statistically significant difference between the two viscosities for the evaluated RC (Table 2).

A previous study is aimed at evaluating microleakage using micro-CT in class 2 bulk-fill composite restorations bonded with self-etch or total-etch techniques [34]. Similar to the present investigation, the authors selected and prepared molars with class 2 cavities and restored them using Filtek Bulk Fill resin. All teeth were thermo-cycled between 5°C and 55°C for 800 cycles. According to their results, the volumetric analysis showed significantly greater leakage in the enamel margins using the self-etch adhesive. It was justified that the micro-CT volumetric evaluation improves the discrimination and represents a more consistent method to assess the microleakage; however, the clinical application of data is still debatable [34]. The present study corroborates with this statement and suggests that further studies should compare both methods to assess the indications and limitations of each.

An in vitro investigation evaluated the microleakage of self-etch and multistep, total-etch adhesive systems [35]. The specimens consisted of class V cavities with coronal margins in enamel and apical margins in dentin. The teeth were submitted to thermocycle for 1000 cycles before the microleakage evaluation in the microscopy. Comparison of the adhesive groups at the enamel margin revealed that the adhesive system showed a significant effect. In contrast, there were no significant differences among the adhesive groups when the dentin margin was evaluated. The results showed significantly less leakage at the enamel margins compared to the dentin margins of the eight adhesive systems tested [35]. The present investigation complements these findings, suggesting that when longer cycles are considered, there is no difference for the microleakage between different restorative protocols.

A systematic review evaluates the marginal integrity of flowable and conventional bulk-fill composite materials placed in class II cavities [36]. The authors highlighted that the marginal integrity in enamel and dentin does not differ significantly between bulk fill composites used for class II restorations. Furthermore, their marginal integrity was comparable to conventional resin composites with incremental techniques. In addition, adhesive system containing a total-etch technique and restoration margin located in enamel resulted in better marginal integrity. However, in this study, there was no difference between self-etching and total etching techniques, probably caused by the long-term simulation using 2000 thermocycling.

Complementary to the microleakage evaluation, the bond strength tests are common methods of assessing the effectiveness of a dental adhesive system [37]. A previous report evaluated the bond strength for two adhesive systems, with baseline values and during fatigue stress. A statistically significant difference between the data of self-etching and total-etching adhesive systems was found, with the value for total-etch adhesive systems being 76% of that for self-etching. It was affirmed that the penetration of resin into the enamel surface for the total-etching system was considerably lower than for self-etching, as estimated, but it is inconclusive that this is the singular reason for the described differences in bond strength and fatigue results between both systems.

According to the literature, reduced polymerization shrinkage leads to incomplete polymerization of the composite in a deeper layer; consequently, it compromises the mechanical properties strategies of the resin composite [38]. A weaker material favors the microleakage, as well as several variables such as dimensional change of the restorative material, thermal contraction, polymerization shrinkage, water sorption, and stresses and dimensional changes of the tooth. Reducing the marginal leakage and improving the marginal adaptation involve various factors such as choice or combinations of materials, use of cavity liner or base, cavity design, acid etching and bonding, technique of restoration placement, and different curing strategies [38].

Therefore, in view of the study conducted and the literature surveyed, future in vitro research is still necessary to effectively evaluate the marginal microleakage of class II restorations with regular, fluid, and bulk fill resins.

## 5. Conclusion

On the basis of this in vitro study, it can be concluded that there is no difference in the microleakage between conventional and bulk-fill resins regardless of the material viscosity or adhesive protocol.

## Figures and Tables

**Table 1:** Groups, etching protocol, materials, composition, and application technique.

Group	Etching:	Materials (abbreviations)	Composition^∗^	Application technique
G1	Total-etching	Filtek Z350 XT (Z350)	Silane-treated ceramics, Bis-GMA, Bis-EMA, silane-treated silica, silane-treated zirconia oxide, dimethacrylate diurethane, polyethylene glycol dimethacrylate, TEGDMA, BHT	Oblique increments of 2 mm in thickness
G5	Self-etching			
G2	Total-etching	Filtek Z350 XT flow (ZF)	Silane-treated ceramics, substitute dimethacrylate, Bis-GMA, silane-treated silica, TEGDMA, ytterbium fluoride, functionalized dimethacrylate polymer, and titanium dioxide	Oblique increments of 2 mm in thickness
G6	Self-etching			
G3	Total-etching	Filtek One Bulk Fill (FBP)	Silane-treated zirconia/silica filler, UDMA, silane-treated zirconia, ytterbium fluoride, DDDMA, silane-treated zirconia, water, AFM-1 monomer, ERGP-DMA, curing agents, stabilizers, dyes	Increments of 5 mm in thickness without conventional resin coating
G7	Self-etching			
G4	Total-etching	Filtek Bulk Fill Flow (FBF)	Ceramics treated with silane, UDMA, substituted dimethacrylate, Bis-EMA, ytterbium fluoride, Bis-GMA, benzotriazole, TEGDMA, and ethyl 4-dimethylaminobenzoate.	Increments of 4 mm in thickness and coverage with conventional resin
G8	Self-etching			

**Table 2:** Mean values and standard deviations of the percentage of microleakage.

Groups	Etching mode	
	TE	SE
Z350	19.96 (22.74)	28.34 (11.61)
ZF + Z350	19.52 (12.11)	21.84 (10.79)
FBP	21.32 (14.78)	28.80 (21.01)
FBF + Z350	32.74 (23.20)	28.74 (21.92)

Abbreviations: TE: total etching; SE: self-etching; Z350: Filtek Z350 XT; ZF: Filtek Z350 XT Flowable; FBP: Bulk Fill Posterior; FBF: Filtek Bulk Fill Flow.

**Table 3:** ANOVA two-way according to etching mode and RC (95%).

Source	Type III sum of squares	Gl	Medium square	*F*	Sig.
Corrected model	4661.139	7	665.877	2.019	0.055
Intercept	125003.283	1	125003.283	378.958	0.000
RC	2604.074	3	868.025	2.631	0.051
Etching	620.414	1	620.414	1.881	0.172
RC ^∗^ etching	1231.881	3	410.627	1.245	0.295
Error	63333.158	192	329.860		
Total	195385.921	200			
Total corrected	67994.297	199			

## Data Availability

The data that support the findings of this study are available on request from the first or last authors.
